# Threats from urban expansion, agricultural transformation and forest loss on global conservation priority areas

**DOI:** 10.1371/journal.pone.0188397

**Published:** 2017-11-28

**Authors:** Victoria Veach, Atte Moilanen, Enrico Di Minin

**Affiliations:** 1 Department of Biosciences, University of Helsinki, Helsinki, Finland; 2 Department of Geosciences, University of Helsinki, Helsinki, Finland; 3 School of Life Sciences, University of KwaZulu-Natal, Durban, South Africa; Chinese Academy of Forestry, CHINA

## Abstract

Including threats in spatial conservation prioritization helps identify areas for conservation actions where biodiversity is at imminent risk of extinction. At the global level, an important limitation when identifying spatial priorities for conservation actions is the lack of information on the spatial distribution of threats. Here, we identify spatial conservation priorities under three prominent threats to biodiversity (residential and commercial development, agricultural expansion, and forest loss), which are primary drivers of habitat loss and threaten the persistence of the highest number of species in the International Union for the Conservation of Nature (IUCN) Red List, and for which spatial data is available. We first explore how global priority areas for the conservation of vertebrate (mammals, birds, and amphibians) species coded in the Red List as vulnerable to each threat differ spatially. We then identify spatial conservation priorities for all species vulnerable to all threats. Finally, we identify the potentially most threatened areas by overlapping the identified priority areas for conservation with maps for each threat. We repeat the same with four other well-known global conservation priority area schemes, namely Key Biodiversity Areas, Biodiversity Hotspots, the global Protected Area Network, and Wilderness Areas. We find that residential and commercial development directly threatens only about 4% of the global top 17% priority areas for species vulnerable under this threat. However, 50% of the high priority areas for species vulnerable to forest loss overlap with areas that have already experienced some forest loss. Agricultural expansion overlapped with ~20% of high priority areas. Biodiversity Hotspots had the greatest proportion of their total area under direct threat from all threats, while expansion of low intensity agriculture was found to pose an imminent threat to Wilderness Areas under future agricultural expansion. Our results identify areas where limited resources should be allocated to mitigate risks to vertebrate species from habitat loss.

## Introduction

The diversity of life on Earth is currently being lost at unprecedented rates[1]. Driven largely by human population growth and consumption, present day extinction rates are estimated to be about 1,000 times higher than those that would be seen in the absence of human pressure[2]. Habitat loss and fragmentation resulting from human appropriation of land for activities such as urban development, agriculture expansion, energy production, etc., is the most serious threat to biodiversity, affecting approximately 85% of all species listed in the IUCN Red List[2–4]. Overexploitation, introduced species, and co-extinctions follow closely behind as the most serious threats to biodiversity[3,4]. Anthropogenic driven climate change poses an additional risk to the persistence of species[5]. Each of these threats poses a great risk to the survival of biodiversity when considered individually. However, there are often several overlapping and interacting stressors present at a location, resulting in a net impact that is greater than the sum of each individual threat[6].

Protected area establishment is one of the key strategies to mitigate threats and curb further loss of biodiversity[7,8]. Identifying important areas for conservation can be carried out either proactively, i.e. prioritizing areas under low threat and preventing stressors by establishing conservation areas prior to the development of a threatening process, or reactively, i.e. mitigating stressors by prioritizing areas that are currently under high levels of threat[9]. Determining which approach is more suitable depends on many complicated factors, including whether the pressures in question really can be stopped or mitigated in the particular area of interest[10,11]. Complementarity-based spatial conservation prioritization (SCP) methods can help achieve effective conservation planning by identifying the most effective network of sites to provide protection for the most species[7,12], while also balancing biodiversity needs with competing land-uses that threaten biodiversity[13,14]. For an SCP planning project to be successful, several types of information is needed, including reliable information about the distributions of the biodiversity features present, distributions of processes threatening biodiversity and competing for land use, and costs[8].

Many studies about threats have been quantitative assessments of the number and proportion of species populations declining due to different threats (habitat loss, disease, non-native species invasions, pollution, etc.) for a country[15–18], a habitat or realm[19,20], or a single taxonomic group[21,22]. Others studies developed spatially explicit maps showing the location and intensity of threats to biodiversity. These maps have been made at variable geographic scales from regional[23] and continental[18] scale to global terrestrial[24], marine[25,26], and coral reef[27] ecosystems. However, global information on the spatial distribution of threats is often missing or inaccurate[28].

Here, we consider three prominent threats to biodiversity: residential and commercial development, agriculture, and forest loss. These stressors threaten the highest number of species of the twelve IUCN threat categories[4,28], and they represent three primary drivers of habitat loss, one of the leading causes of biodiversity decline[3]. We focus on these three threats due to the availability of reliable broad-scale data for terrestrial ecosystems. We first explore how global priority areas for conservation would differ when each threat is individually accounted for in prioritization. We then assess the potential for simultaneously including the three threats in a single priority area network. Finally, we assess four well-known global conservation priority area schemes with respect to overlap with these three prominent threats.

## Methods

### Biodiversity features

We based our analysis on species range maps for mammals, birds, and amphibians fully assessed in the IUCN Red List[4]. Range maps for birds were obtained from BirdLife International’s Data Zone page[29] and range maps for mammals and amphibians were obtained from the IUCN Red List web site[4]. We omitted reptiles because at the present time a large proportion of reptiles have not been sufficiently assessed and reliable range distribution data is not yet available[4]. Due to the nature of the threats investigated, the analysis was applied to terrestrial areas only. Therefore, only terrestrial species were selected for our analyses.

Species range maps from the IUCN Red List and BirdLife International were available as GIS polygons covering the known or inferred areas where species occur[4]. Prior to spatial prioritization, all range maps were converted to a latitude/longitude coordinate system and rasterized to global high resolution grids of 1 degree, or 10 km at the equator. We assigned pixel values in the rasterized maps as presence/absence according to the certainty of species presence in the polygon reported by the IUCN. Areas reported by the IUCN as extant and probably or uncertainly extant were assigned a value of 1 in the rasterized maps, while all other categories were assigned a value of 0.

We included a condition transformation[30,31] in each analysis. The condition layer was based on the land-use model created by van Asselen and Verburg (2013)[32] and reflects land-use and habitat quality for the year 2000. Each land-use class in the model was assigned a value to reflect its naturalness, with 0 indicating a land-use that is totally unsuitable for biodiversity and 1 indicating pristine conditions. The specific values used in the condition layer are defined in Pouzols et al. (2014)[33] and here they are used as surrogates of agricultural threat level (Table 1). Basically, the condition transformation adjusts the distribution of each feature by multiplying the pixel values in each species layer by the values in the condition layer. The condition layer helps avoid setting priority areas in locations that have become unsuitable for biodiversity due to broad-scale land-use conversion and subsequent habitat loss by effectively cutting out already transformed areas from the species range maps, which may have not been fully up-to-date with respect to land conversion.

**Table 1:** The van Asselen and Verburg (2013) agriculture classes, habitat condition layer value, and the threat intensity levels used here. We use ‘intensity’ qualitatively here to explore how the assumed severity of the impact on the natural system is distributed within the broader threat category.

Verburg Land System	Condition Layer Value	Intensity Level
All forms of intensive and medium intensive cropland	0.2–0.3	High
Extensive cropland and intensive/medium intensive mosaic cropland	0.4–0.6	Medium
Extensive mosaic cropland	0.7–0.8	Low

### Spatial conservation prioritization

**Fig 1:**
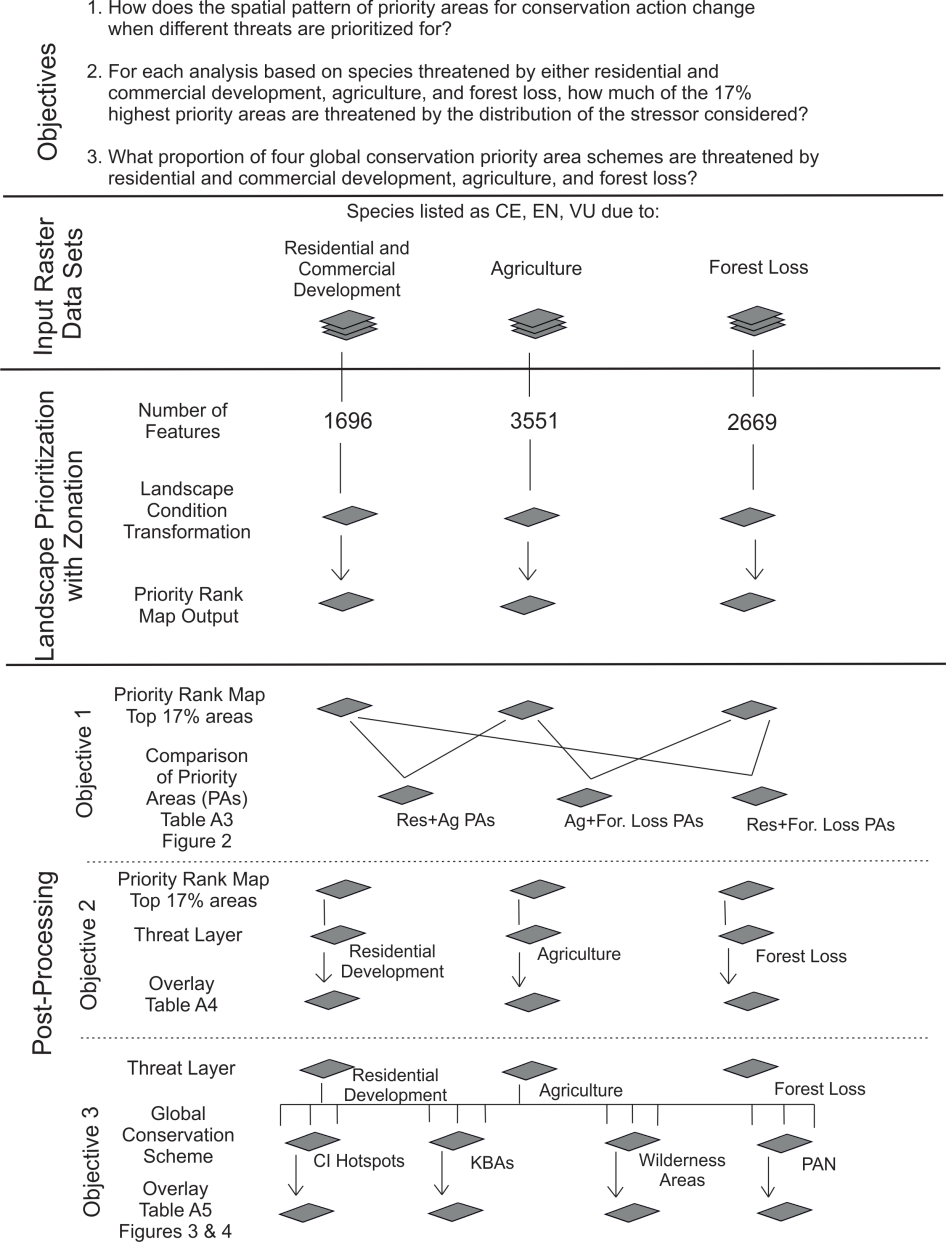
Schematic of the study design.

Fig 1 summarizes the flow of computations in this study. We identified priority areas for conservation action using the Zonation software v. 4.0 for spatial conservation prioritization[31,34]. Zonation implements computational methods for broad-scale spatial landscape prioritization using large sets of grid-format maps about biodiversity features, costs, and threats as well as additional ecological information about, e.g., connectivity effects[31,33–35]. Each Zonation prioritization begins from the full landscape and creates a hierarchical priority ranking by iteratively ranking and removing the grid cells which cause the least marginal loss in aggregate conservation value[31,33,36,37]. As grid cells are ranked and removed during a prioritization, the landscape-level representation of each feature (i.e. species, habitats) declines. Zonation tracks this loss of representation and uses it to balance the relative importance of the remaining occurrences of features[13,31,38], allowing Zonation to retain balanced representation for all features throughout the prioritization. Zonation has multiple options for how marginal loss of conservation value is aggregated across species. In this study, we used the additive benefit function (ABF)[39], which effectively uses feature-specific species area curves to minimize aggregate extinction risk[13,31].

We conducted a separate prioritization for species coded by the IUCN as threatened by residential and commercial development; agriculture expansion; and forest loss. Each prioritization was based only on the species that are threatened (critically endangered, endangered, or vulnerable) in the IUCN Red List[4]. A threat-specific analysis considering only the species that are at risk of extinction due to each threat allowed us to locate the areas where biodiversity is most susceptible to each threat. In total, the number of terrestrial vertebrate species included in each analysis was 1,696 for residential and commercial development, 3,551 for agriculture expansion, and 2,669 for forest loss.

### Overlay analyses with threat layers

We conducted post-processing overlay analyses in R version 3.2.3[40], using the raster[41] and zonator[42] packages, and in ArcGIS v10.1[43]. In this analysis, we assessed the extent of overlap between the three sets of the 17% highest priority areas identified in this study and the spatial threat distribution. We did the same for four global conservation schemes, namely Key Biodiversity Areas[44], the Biodiversity Hotspots[45,46], the World Wilderness Areas[47], and the global Protected Area Network[48]. We included three types of threat in the overlay analysis: residential and commercial development, agriculture expansion, and forest loss.

As threat layers, we used the 2040 expected distributions of residential and commercial development and agriculture expansion created by the van Asselen and Verburg (2013)[32] land systems model. We were primarily interested in expected changes in the distributions of threats, which is why we modified the layers to show only the expected threat distribution expansion by year 2040. Hence, we clipped out from the threat distribution maps the areas classified as residential and commercial development and agriculture in 2000. For forest loss, we used the forest loss layer created by Hansen et al. (2013)[49]. This layer shows the proportion of forest loss between the years 2000 and 2012 from both anthropogenic forest loss through deforestation, as well as natural forest loss via events such as stand replacing fire or blow down during storms. In contrast to the predictive agriculture and urban expansion layers included, the forest loss information included here is retrospective due to a lack of reliable estimates regarding future forest loss. While this presents a discrepancy in the type of data used in this study, our overall objective was to explore the spatial relationships between the distributions of threats and priority areas. We aggregated this layer to 10km resolution at the equator using ArcGIS v10.1[43] to be consistent with the other grid data layers included in this study.

For assessing the extent to which the three sets of priority areas and four global conservation schemes are threatened, we broke agriculture and forest loss into low, medium, and high intensity classes by using tertiles (Table 2). Tertiles are any of the two points that divide an ordered distribution into three parts, each containing a third of the population. In this case, splitting the two layers into 3 categories each using tertiles allowed us to simplify the interpretation and the presentation of the results. Moreover, the objective here was to simply calculate the overlap between the priority and threat grid cells and not to carry out more sophisticated correlation analyses. Thus, the levels of intensity used in this study are purely qualitative and were used to explore how the different level of assumed impact on the natural habitat (low habitat loss ranging to complete land use conversion) were distributed within the broader threat classifications. The agriculture classes were based on the original land system classes developed by van Asselen and Verburg[32] and the values given to each in the condition layer (Table 1). Forest loss intensity classes were based on the amount of the original forest that has been lost divided into tertiles (Table 2). For residential and commercial development, we had just two classes—urban and periurban—as these were the only two residential classes classified in the land systems model of van Asselen and Verburg[32].

**Table 2:** Forest loss threat levels used here. We use ‘intensity’ qualitatively here to explore how the amount of forest loss is distributed within the broader threat category.

Amount of original forest lost	Forest Loss Threat Intensity
5.4–85.5%	High
1.0–5.4%	Medium
0–1.0%	Low

## Results

**Fig 2:**
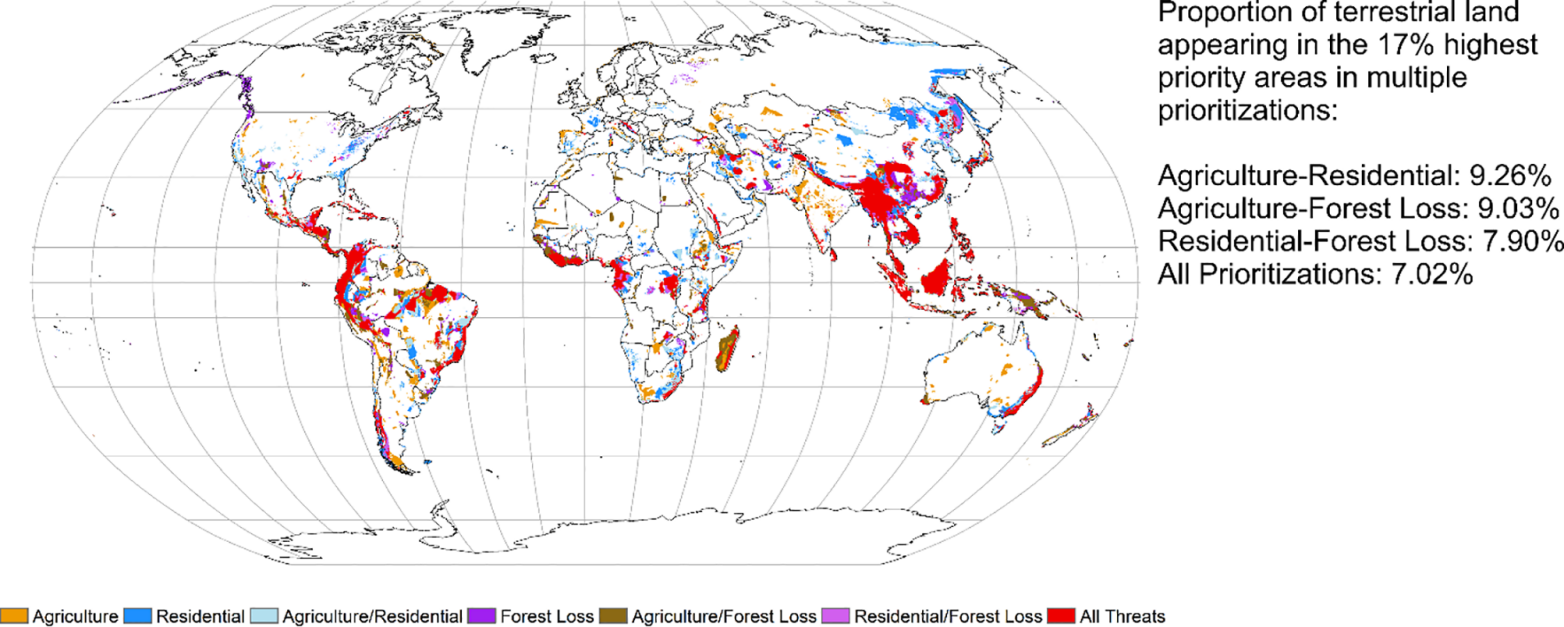
Highest ranking 17% of priority areas when based on species threatened by agriculture (yellow), residential and commercial development (light purple) and forest loss (dark green). Areas in dark purple appear in the highest 17% priority areas in both agriculture and residential prioritizations. Areas in dark green show areas that are ranked in the highest 17% priority areas in the prioritizations based on agriculture and forest loss, and areas in blue appear in the highest 17% priority areas in the residential and forest loss priority areas. Areas in red are ranked in the highest 17% priority areas in all prioritizations.

Spatial conservation prioritization for species threatened by a single threat (residential and commercial development, agriculture expansion, and forest loss) resulted in different spatial patterns of global priority areas (Fig 2). For species threatened by agriculture, high priority areas occurred throughout central Asia, India, and Australia, while high priority areas for species threatened by residential and commercial development appeared in eastern Mongolia, north eastern China, and eastern Siberia (Fi. 2). High priority areas for species threatened by forest loss were most prominent in south western China with smaller clusters in South America and western Russia (Fig 2).

A pair-wise comparison using the Jaccard Similarity Index showed that about half of the 17% highest priority areas identified by Zonation for each threat were shared by another set of priority areas. Overlap between agricultural and urban expansion prioritizations was 0.53; overlap between agricultural expansion and forest loss prioritizations was 0.59; and overlap between urban expansion and forest loss prioritizations was 0.51. When all three sets of priority areas were considered in the same analysis, we found that 7.02% of the total terrestrial surface appeared in the top 17% priority areas in all analyses (Fig 2). The areas where the three sets of priority areas overlap are mostly located in the highly biodiverse tropical regions of the world.

Between 43.02% and 46.56% of the highest priority areas identified by Zonation directly overlapped the distribution of at least one of the threats considered here (Table 3). Where the three sets of top 17% priority area sets intersect, 52.14% of the area was directly threatened by either agriculture, residential and commercial development, or forest loss (Table 3). Forest loss affected the most area within the priority areas, ranging from 31.25% to 36.82% of the individual top 17% priority areas and 41.91% of the area where the three sets of priority areas intersect (Table 3). Agriculture expansion, particularly high intensity agriculture, also threatened a considerable amount of area in each of the priority areas. In total, agriculture threatened approximately 22% of each individual priority area and 24.21% of the area where the three sets of priority areas intersect (Table 3). Residential and commercial development threatened around 4% of each set of priority areas, with most of the total area threatened by expansion and development of peri-urban areas (Table 3).

**Table 3:** Proportion of top 17% priority areas that are directly threatened by each of the threats. Intensity is used qualitatively to explore how the assumed impact on the natural system is distributed within the broader threat classification.

Threat	Top 17% Priority Area			
	Residential	Agriculture	Forest Loss	3 Priority Area Overlap
Agriculture				
High Intensity	11.35%	10.75%	9.78%	10.94%
Mid Intensity	4.38%	4.00%	4.30%	4.64%
Low Intensity	6.59%	6.51%	7.59%	8.63%
Total	22.32%	21.26%	21.67%	24.21%
Residential				
Urban	0.68%	0.60%	0.58%	0.60%
Peri-urban	3.40%	3.06%	3.11%	3.61%
Total	4.08%	3.66%	3.69%	4.21%
Forest Loss				
High Loss	18.98%	18.63%	21.80%	25.00%
Mid Loss	12.85%	12.62%	15.01%	16.92%
Low Loss	8.67%	8.40%	9.83%	10.66%
Total	31.82%	31.25%	36.82%	41.91%
Total from all threats combined	44.31%	43.02%	46.59%	52.14%

Of the four global conservation schemes considered in this study, the Biodiversity Hotspots had the greatest proportion of their total area under direct threat (Table 4). Approximately 44% of the total area of Hotspots was threatened by at least one of the threats included here, compared to approximately 24% of KBAs, 15% of PAs, and 7% of Wilderness areas (Table 4). Forest loss threatened the largest area of each scheme, affecting between 6.20% of Wilderness areas to 30.58% of Hotspots (Table 4). Agriculture expansion also threatened a considerable proportion of each scheme, ranging between 1.56% of Wilderness areas to 22.47% of Hotspots. Residential and commercial development threatened the least amount of area in all global conservation schemes (Table 4).

**Table 4:** Proportion of global conservation schemes that fall within the top 17% Zonation priority area and are directly threatened by at least one of agriculture, residential and commercial development, or forest loss. Numbers in parentheses show the total proportion of the area threatened in each scheme. Intensity is used qualitatively to explore how the assumed impact on the natural system is distributed within the broader threat classification.

	CI Hotspots	Wilderness	PA (all)	KBA
Agriculture				
High Intensity	6.20% (12.30%)	0.18% (0.47%)	1.24% (2.43%)	3.28% (6.87%)
Mid Intensity	2.10% (3.39%)	0.08% (0.36%)	0.55% (1.51%)	1.28% (2.77%)
Low Intensity	4.43% (6.77%)	0.20% (0.73%)	0.95% (2.08%)	1.82% (3.25%)
Total	12.73% (22.47%)	0.46% (1.56%)	2.74% (6.03%)	6.39% (12.89%)
Residential				
Urban	0.26% (0.46%)	0.00% (0.06%)	0.06% (0.11%)	0.09% (0.22%)
Peri-urban	2.06% (3.78%)	0.02% (0.01%)	0.38% (0.74%)	0.67% (1.65%)
Total	2.32% (4.24%)	0.03% (0.05%)	0.44% (0.85%)	0.77% (1.87%)
Forest Loss				
High Loss	12.43% (18.14%)	1.10% (4.38%)	2.87% (6.54%)	5.36% (8.71%)
Mid Loss	8.71% (12.43%)	0.50% (1.81%)	1.86% (4.03%)	3.44% (5.66%)
Low Loss	5.62% (8.63%)	0.36% (1.60%)	1.54% (3.52%)	2.89% (5.02%)
Total	21.14% (30.58%)	1.60% (6.20%)	4.73% (10.57%)	8.80% (14.37%)
Total from all threats combined	34.49% (43.98%)	2.66% (7.25%)	9.68% (14.52%)	18.15% (23.96%)

Expansion of high intensity agriculture and areas that have experienced a high amount of forest loss (5.4%-85.5% loss of original forest cover) contributed most to the overall agriculture and forest loss threat in all schemes. The one exception to this was Wilderness areas, where expansion of low intensity agriculture contributed the most area to the total threat of growing agriculture activities. In Hotspots and KBAs, high intensity agriculture accounted for more than half of the total area threatened by agriculture expansion while areas that have lost a high amount of original forest account for up to 60% of the total area that has experienced forest loss.

**Fig 3:**
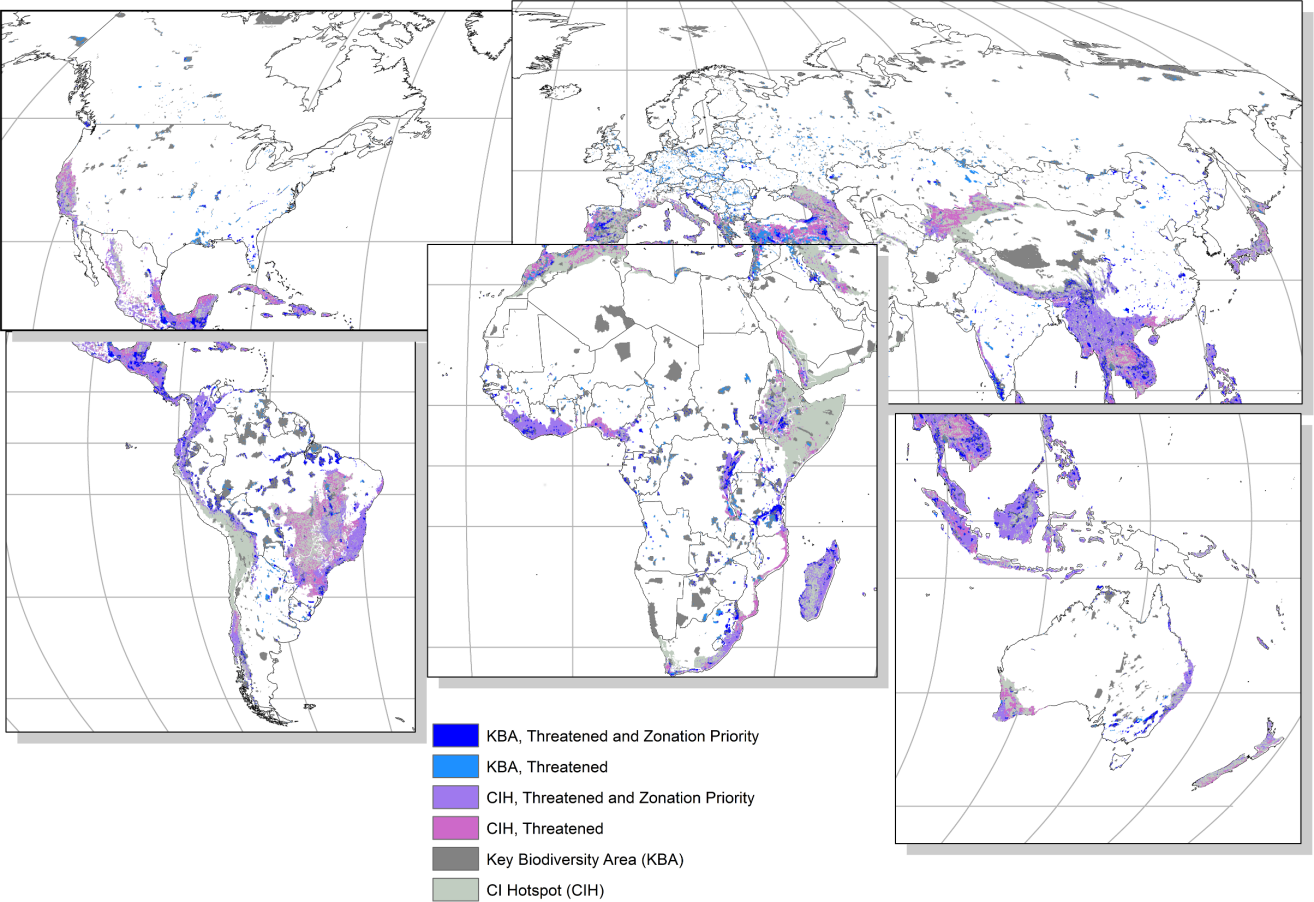
Key biodiversity areas and conservation international hotspots that are threatened by at least one of residential and commercial development, agriculture expansion, or forest loss (labelled ‘Threatened’) and fall within the highest ranking 17% priority areas from all three analyses (labelled ‘Threatened and Zonation Priority’).

**Fig 4:**
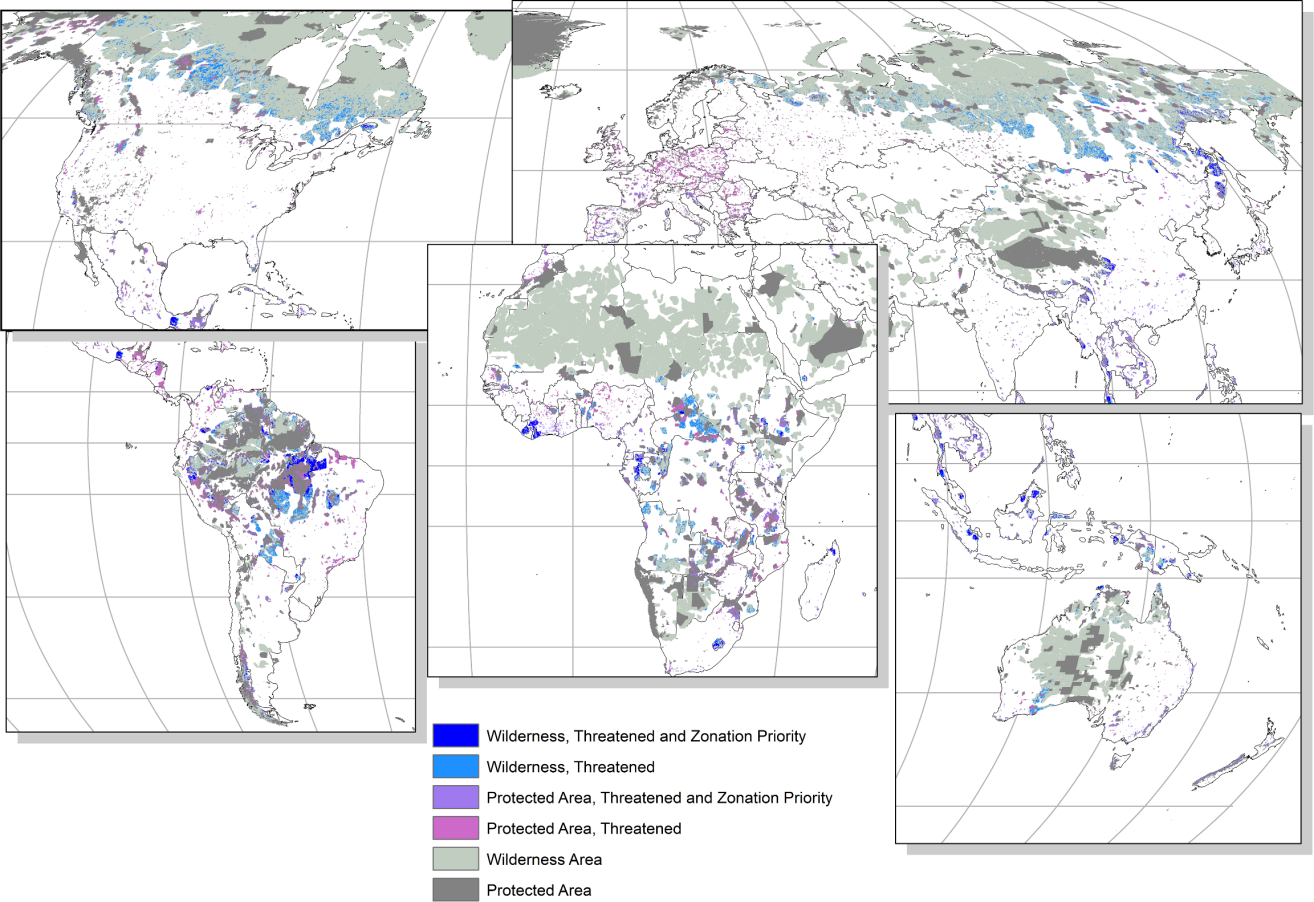
Wilderness areas and protected areas that are threatened by at least one of residential and commercial development, agriculture expansion, or forest loss (labelled ‘Threatened’) and fall within the highest ranking 17% priority areas from all three analyses (labelled ‘Threatened and Zonation Priority’).

A large proportion of Hotspots, PAs, and KBAs that are directly threatened by agriculture expansion, residential and commercial development, and forest loss were also ranked within the top 17% priority areas in Zonation (Figs 3 and 4). Of the total area impacted by each threat considered here, between 55% and 69% of Hotspots, 45–52% of PAs, and 41–62% of KBAs also appeared in the 17% highest ranked areas from at least one of the Zonation priority rankings. In all of the global conservation schemes included here, the areas that were both directly threatened and a Zonation priority area occurred mostly in the tropical regions, South East Asia, and along the South-eastern regions of Africa. Some threatened and Zonation priority area KBAs and PAs also occurred in Europe, North America, and Australia, and some threatened and Zonation priority area Wilderness areas were found in the eastern parts of Siberia (Figs 3 and 4).

Although quite a large extent of the conservation schemes that were threatened were also ranked as high priority areas in Zonation, there were still many areas of the conservation schemes that were threatened but not ranked as a Zonation priority area for the conservation of vertebrate species (Figs 3 and 4, Table 4).

## Discussion

Anthropogenic threats to biodiversity take many different forms, originate from many different activities, and are globally pervasive. There is not one single place left on earth that is not experiencing at least one threat originating from the actions of humans[24]. In fact, most places are suffering from multiple, often very different threats[24,26]. The results of this study start building an understanding of where threats occur and how their spatial patterns relate to both other threats and to priority areas for conservation—an understanding that is helpful for allocating resources to mitigate such threats.

The overlap of the threat-specific priority areas found in this study suggests that there is potential for protecting species from multiple threats with well-planned and placed conservation areas, especially in the highly biodiverse tropics. In this study, we identified priority areas for species at risk due to residential and commercial development, agriculture, and forest loss—all of which are strong drivers of habitat loss[49–52]. Priority areas based on species at risk for extinction due to threats driving other processes, such as over-exploitation, invasive species, or pollution, might show different spatial patterns and require strategies other than protection in reserves.

In Zonation it is possible to include areas valuable to alternative land uses as places to avoid giving high conservation priority to[13,14]. We did not use this function for these prioritizations. Rather, our prioritizations were based solely on the species at risk due to the specific threats included here. After identifying the priority areas, we assessed how much the threat layers overlap the priority areas (Fig 2; Table 3). Our aim was to assess how much direct threat each set of priority areas faces. Our results indicate that potential impacts of different threats to conservation priority areas vary significantly. For example, residential and commercial development are smaller threats. This suggests that it may be possible to avoid conflict with this land use. On the other hand, forest loss and agricultural expansion are much larger threats and might require reactive approaches to stop them[9].

An important question with threats is whether they are stoppable, unstoppable, or partially stoppable[10,11]. Climate change, for example, is unstoppable from the local perspective: its effects will depend on global efforts that cannot significantly be influenced locally. It is best to stay away from locations that face unstoppable threats. In this study, we took the attitude that we are working with stoppable threats: the locations of urban expansion, agricultural expansion, and forestry operations can be decided by local administrative action. Hence, these threats are stoppable (at least partially), and looking for threatened areas therefore makes sense when targeting conservation actions.

An added difficulty of planning for threats is the widespread lack of spatial data showing the location of individual threats, making it difficult to fully understand the degree of threat present at a location[28]. Our study addresses this by locating threat-specific priority areas for conservation of species threatened by three dominant threats and exploring the spatial relationships between the three resulting sets of priority areas. The high amount of spatial overlap among the sets of priority areas (Fig 2) indicates that there may be potential for mitigating multiple threats with well-planned and placed conservation areas. However, we could only include three threats in this study, and we thus cannot fully understand where to place conservation areas that would best mitigate all threats together.

Our results also highlight that global conservation schemes, such as biodiversity hotspots and key biodiversity areas, are under threat from habitat loss. While strict protection remains crucial to enhance the persistence of the threatened species in these areas, it is important that sound resources are invested for protected area management[53]so that many of the protected areas that are and will be created are more than ‘paper’ parks[54]. As many of the protected areas are found in the global south, international donors should also play a key role in making sure resources for conservation are adequate[55]. Other interventions, targeting some of the drivers of habitat loss, including careful land use planning, will be needed to safeguard biodiversity and the services it provides to humans.

Identifying priority areas for conservation actions to stop threats is a cornerstone of systematic conservation planning. Stopping habitat loss is challenging, but international policy, such as the UN Sustainable Development Goals, provide an important framework to stop threats and prevent the perverse mechanisms of biodiversity loss by humans. In this study, we highlight areas where resources should be allocated to prevent habitat loss. Our analyses are based on threats that are driving habitat loss only and that can be stopped with strict protection and adequate management. Future studies should focus on other threats, such as overharvesting, for which no information on the most threatened areas is available[56]. Future studies should also include other species from comprehensively assessed taxonomic groups and not only vertebrates[57].
